# Alterations in Motor Cortical Representation of Muscles Following Incomplete Spinal Cord Injury in Humans

**DOI:** 10.3390/brainsci8120225

**Published:** 2018-12-16

**Authors:** Hunter J. Fassett, Claudia V. Turco, Jenin El-Sayes, Aimee J. Nelson

**Affiliations:** Department of Kinesiology, McMaster University, Hamilton, ON L8S 4L8, Canada; fassethj@mcmaster.ca (H.J.F.); turcocv@mcmaster.ca (C.V.T.); elsayej@mcmaster.ca (J.E.-S.)

**Keywords:** TMS, SCI, motor cortex, somatotopy, reorganization, neurological injury

## Abstract

(1) Background: The primary motor cortex (M1) experiences reorganization following spinal cord injury (SCI). However, there is a paucity of research comparing bilateral M1 organization in SCI and questions remain to be answered. We explored the presence of somatotopy within the M1 representation of arm muscles, and determined whether anatomical shifts in these representations occur, and investigated the symmetry in organization between the two hemispheres.; (2) Methods: Transcranial magnetic stimulation (TMS) was used to map the representation of the biceps, flexor carpi radialis and abductor pollicis brevis (APB) bilaterally in nine individuals with chronic incomplete cervical spinal cord injury and nine aged- and handed-matched uninjured controls. TMS was delivered over a 6 × 5 point grid that encompassed M1 using an intensity specific to the resting motor threshold for each muscle tested.; (3) Results: Results indicate that, compared to controls, muscle representations in SCI are shifted medially but preserve a general somatotopic arrangement, and that territory dedicated to the APB muscle is greater.; (4) Conclusions: These findings demonstrate differences in the organization of M1 between able-bodied controls and those with incomplete cervical SCI. This altered organization may have future implications in understanding the functional deficits observed in SCI and rehabilitation techniques aimed at restoring function.

## 1. Introduction

Human studies have used transcranial magnetic stimulation (TMS) to non-invasively map the location and spatial size of cortical muscle representations. This is achieved by delivering single pulses of TMS to primary motor cortex (M1) and recording the motor evoked potential (MEP) from target muscles [1,2]. Common measures used to characterize these motor maps include area of the cortical territory and center of gravity (CoG). Using TMS mapping, we can explore the spatial territory allocated to specific muscles and the location of the representation within the general homuncular distribution. This technique is extremely useful for characterizing and understanding the cortical reorganization that occurs following neurological injury.

Spinal cord injury (SCI) involves neurological trauma to ascending and descending neural pathways resulting in impairments in sensation and motor control. Reorganization occurs in M1 as a consequence of alterations to motor pathways and proprioceptive afference [3]. In SCI, regaining motor function is accompanied by reorganization within M1. During the first year following injury, functional activity within M1 is positively correlated with upper limb function improvement [4]. Similarly, motor practice improves hand function and expands the M1 representation of a thumb muscle [5]. In complete SCI, cortical territory is expanded for spared muscles rostral to the level of injury [1,6,7] but does not appear to change following incomplete SCI [8,9]. Similarly, somatotopic shifts occur in SCI [10] but not always [1,6,7,8,9]. Notably, previous investigations have been performed on SCI groups with varying injury severity and without consideration of hand dominance, although multiple studies have shown interhemispheric differences in motor cortex organization based on laterality [11,12,13,14,15].

To date, there is a paucity of research examining bilateral M1 reorganization in SCI. Previous TMS studies have mainly investigated muscle representations in one hemisphere following SCI [5,6,7,8,9,10], and therefore it is unknown whether reorganizational changes exist within both hemispheres and whether or not symmetry between hemispheres exists. Therefore, important unanswered questions regarding somatotopy, expansion, and symmetry remain. These questions are important since quantifying M1 map characteristics in SCI may be useful for predicting and monitoring functional recovery. The goal of the present study was to provide a detailed characterization of bilateral M1 in a group of incomplete SCI participants with similar injury severity relative to uninjured, age- and handed-matched controls. For individuals with cervical SCI, regaining arm and hand function is considered a top priority [16] and the present work aims to understand the M1 reorganization of upper limb muscles. Our data indicate that, in SCI, reorganizational changes occur that include expansion of the hand muscle representation and a medial shift in the representation of muscles relative to controls. However, SCI demonstrate a preserved general somatotopic arrangement, similar to uninjured controls.

## 2. Materials and Methods

### 2.1. Participants

Eighteen individuals, nine SCI (46.1 ± 12.4 years) and nine age-, handed-, and sex-matched able-bodied controls (40.3 ± 13.5 years) participated in the study (T _(16)_ = 0.941, *p* = 0.360) (Table 1). SCI participants presented with chronic (defined as a minimum of two years post-injury) incomplete injury at the level of C3–C8 with American Spinal Injury Association (ASIA) score C [17]. Participants were tested in their regularly medicated state (Table 1). Handedness was determined using a modified handedness questionnaire [18]. The study conformed to the declaration of Helsinki and was approved by the Hamilton Integrated Research Ethics Board (HIREB #13-112) on 18 March 2013. All individuals provided written consent prior to participation.

### 2.2. Electromyography (EMG)

EMG was recorded bilaterally from biceps brachii (BB), flexor carpi radialis (FCR), and abductor pollicis brevis (APB) muscles using surface electrodes (9 mm diameter Ag-AgCl). Recordings from BB and FCR used a bipolar montage over the muscle belly. EMG from APB was measured via a monopolar configuration with one electrode placed over the muscle belly and the other over the metacarpal-phalangeal joint. All EMG recordings were band-pass filtered between 20 Hz and 2.5 kHz, amplified 1000× (Intronix Technologies Corporation Model 2024F, Bolton, ON, Canada), and an analog-to-digital interface was used to digitize recordings at 5 kHz (Power1401, Cambridge Electronics Design, Cambridge, UK). 

### 2.3. M1 Mapping Protocol

**Figure 1:**
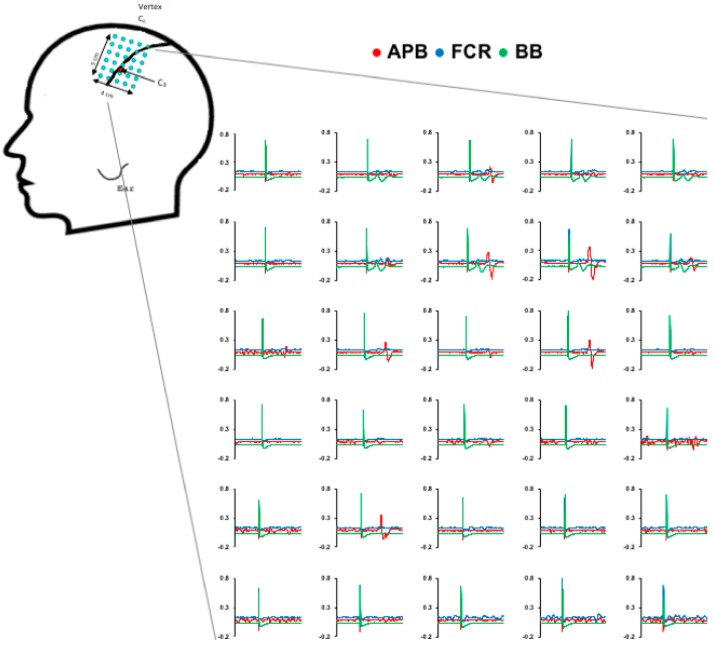
Sample data from a spinal cord injury (SCI) participant for generation of cortical territories. Raw traces are shown for each muscle at each grid point.

All TMS delivery was done with two customized 50 mm diameter figure-of-eight branding coils each connected to a Magstim Plus stimulator (Magstim, Whitland, UK), one coil per hemisphere. Figure 1 displays a sample of the 6 × 5 point grid space used (1 cm spacing). Using Brainsight Neuronavigation (Rogue Research, Montreal, QC, Canada), the grid was centered on a location 1.5 cm medial to C3/C4 (International 10–20 system). The grid was rotated 45 degrees from the sagittal plane to approximate the orientation of the central sulcus. This grid included 5 points of stimulation from anterior-to-posterior to encompass the precentral gyrus (M1) that is 3.5 cm [19] with minimal coverage extending to premotor or parietal cortices. The grid included 6 points of stimulation from medial-to-lateral and occupied the extent of precentral gyrus as described anatomically [19]. During all TMS, the coils were always oriented 45 degrees from the sagittal plane to induce a posterior to anterior current. Following grid placement, the motor hotspot was determined for each muscle by delivering a single TMS pulse at each grid point at an intensity of 80% of the maximum stimulator output (MSO). The location in the grid whereby the largest peak-to-peak MEP amplitude was measured for the target muscle was defined as the motor hotspot (i.e., location for APB, location for FCR, etc.). If no hotspot was found at this intensity, the grid was probed at 100% MSO. TMS pulses were delivered.

### 2.4. Resting Motor Threshold

Resting motor threshold (RMT) was obtained at the motor hotspot for each muscle. RMT was defined as the percentage of the MSO that produced a MEP of ≥50 µV peak-to-peak amplitude in 5 out of 10 consecutive trials [20]. This procedure was performed for each muscle in both hemispheres. The TMS intensity used for mapping was set to a suprathreshold intensity corresponding to 120% RMT specific to the muscle tested (Figure 1, right). Maps were generated from each hemisphere by delivering three TMS pulses sequentially to each grid point with an interstimulus interval of 5 seconds (i.e., a total of 9 stimuli were delivered to each grid point; 3 at 120% RMT for BB, 3 at 120% RMT for FCR, and 3 at 120% RMT for APB).

### 2.5. Analysis of M1 Maps

Individual MEPs were included in analysis if the following conditions were met: (1) the EMG amplitude during a 30 ms pre-stimulus window did not exceed the mean plus two standard deviations of the EMG noise across all trials within a given map, and (2) the MEP had an amplitude ≥50 μV. Any trials that did not meet these criteria were removed from further analysis [2,21]. Subsequently, the peak-to-peak MEP amplitude from the remaining MEPs at each grid point were averaged [14]. After computing the averaged MEP response values at each grid point, the size of the cortical territory dedicated to a given muscle (in cm^2^) only included grid points that elicited MEPs exceeding 10% of the maximum averaged MEP response within a given map to account for variable responses in the boundaries of the representation [11,21,22]. CoG for each map was calculated to obtain the amplitude weighted center in the anterior-to-posterior (CoG _(AP)_) and medial-to-lateral (CoG _(ML)_) orientations using the following formula:

$$\mathrm{CoG}=\frac{\sum^{}\alpha_{i}X_{i}}{\sum^{\text{​}}\alpha_{i}}$$

where α_i_ is the average amplitude at a given grid point and X_i_ is the point’s position in the grid [14].

### 2.6. Statistical Analysis

Each dependent measure was assessed for normality (Shapiro-Wilks) and outliers. RMT, cortical territory (cm^2^), CoG, and motor hotspot were compared using three-way ANOVAs with between-subject factor GROUP (2 levels: SCI, Control) and within-subject factors MUSCLE (3 levels: APB, FCR, BB) and HEMISPHERE (2 levels: Dominant, Non-Dominant). For any non-normal data, the data was ranked, and a Conover’s ANOVA was performed (Conover et al., 1981). Significant main effects and interactions were assessed using two-tailed *t*-tests. Results exceeding a level of significance of *α* < 0.05 are reported. 

## 3. Results

**Figure 2:**
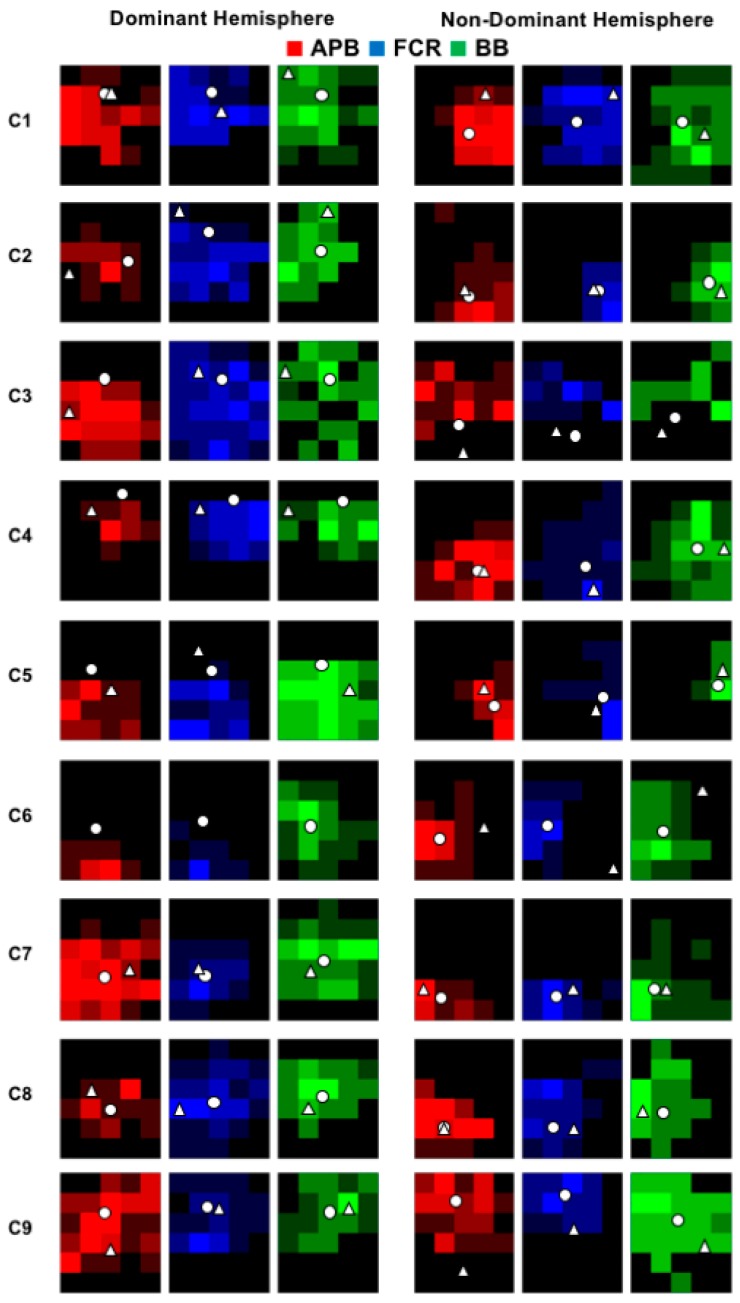
Individual cortical territory plots for each hemisphere and muscle for the control group. Pixel maps represent the size of the cortical territories and the white circle indicates the location of the center of gravity (CoG).

**Figure 3:**
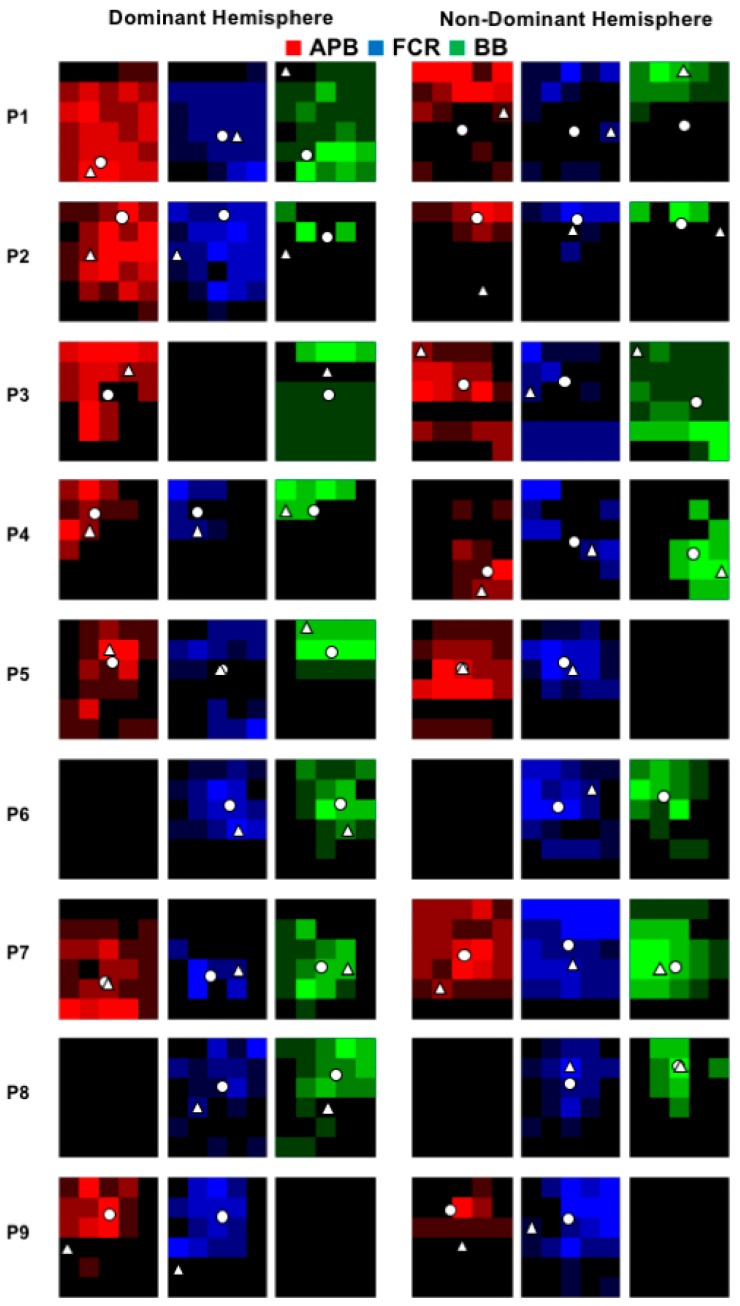
Individual cortical territory plots for each hemisphere and muscle for the SCI group. Pixel maps represent the size of the cortical territories and the white circle indicates the location of the CoG.

**Figure 4:**
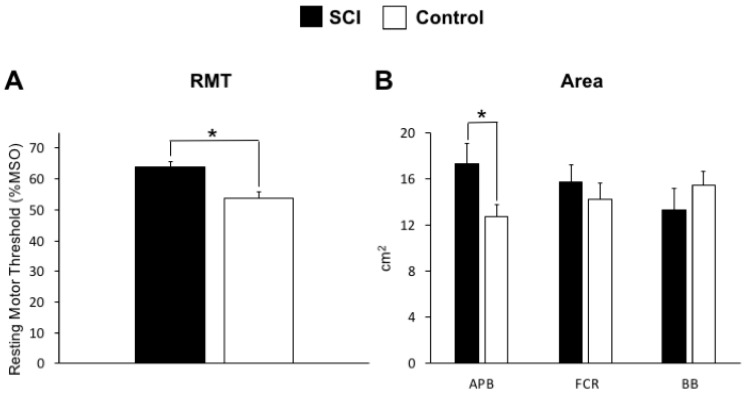
(**A**) The main effect of GROUP revealed that the SCI group had larger higher resting motor threshold (RMT) % maximum stimulator output (MSO) values in the relative to controls (*p* = 0.0289). (**B**) The abductor pollicis brevis (APB) muscles demonstrate larger map areas (defined as the number of active grid points) in the SCI group relative to controls (*p* = 0.030). * indicates a significant difference between groups.

All participants successfully completed the experiment and individual data is displayed in Figure 2 (control group) and 3 (SCI group). In the SCI group, data was not obtained from bilateral APB muscles in two participants, from the non-dominant FCR muscle in one participant, and bilateral or the non-dominant BB muscles from two participants (Figure 3). In these instances, TMS set to 100% MSO did not evoke MEPs in the resting muscle. Assessment of RMT revealed a main effect of GROUP (F _(1,16)_ = 5.76; *p* = 0.0289), plotted in Figure 4A, indicating a higher RMT in SCI versus controls (*p* = 0.0289). 

The group-averaged cortical territory is shown in Figure 4B (cm^2^ with standard error). Three-way ANOVA demonstrated a GROUP×HEMISPHERE×MUSCLE interaction (F _(2,22)_ = 8.265; *p* = 0.002) whereby the SCI group had a larger muscle representation for the APB muscle compared to controls (*p* = 0.030). Post-hoc statistical analyses indicated no differences in cortical territory between hemispheres and can be seen in the individual data for controls (Figure 2) and SCI (Figure 3).

**Figure 5:**
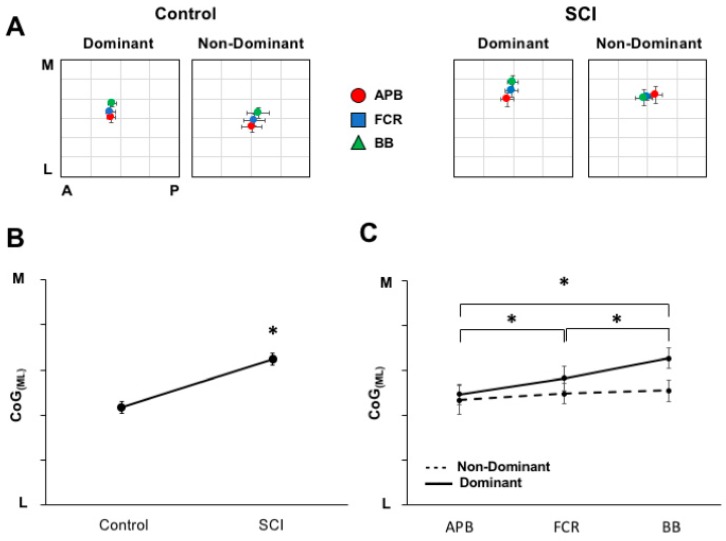
Differences in cortical territory somatotopy between hemispheres and groups. (**A**) The pooled CoG across muscles for each group for each hemisphere. (**B**) The medio-lateral CoG positions across groups demonstrates the main effect of group. The SCI group shows medially shifted muscle representations compared to controls (*p* = 0.041). (**C**) Differences across hemispheres and muscles were seen such that a somatotopic progression is observed in the dominant hemisphere with biceps brachii (BB) located medial to flexor carpi radialis (FCR) (*p* < 0.001) and APB (*p* = 0.01), and FCR is medial to APB (*p* = 0.032). * indicates a significant difference between muscles of the dominant hemisphere.

The group-averaged CoG data (with standard error) for each hemisphere and group are plotted in Figure 5A (left, control group; right, SCI group). For CoG _(ML)_, a main effect of GROUP (F _(1,11)_ = 5.346 *p* = 0.041) (Figure 5B) indicated a medial shift in muscle representation for the SCI group compared to controls. Further, a MUSCLE×HEMISPHERE interaction (F _(2,22)_ = 3.817; *p* = 0.038) (Figure 5C) such that, in the dominant hemisphere only, BB representation was located medial to FCR and APB (*p* < 0.001 and 0.01, respectively), and FCR was located medial to ABP (*p* = 0.032), confirming the general somatotopic distribution. For CoG _(AP)_, no statistical differences between groups, muscles, or hemispheres were observed. In summary, representations in SCI are shifted medial relative to controls. Both SCI and controls demonstrate a general somatotopic organization for muscles of the dominant limb.

## 4. Discussion

The present study examined bilateral cortical organization in individuals with chronic incomplete cervical SCI and uninjured controls. Several novel findings were revealed that provide new information regarding the reorganizational changes that manifest in M1 following SCI. We discuss these findings, their potential mechanisms, and their implications for understanding reorganization following SCI. 

Although motor maps were not obtainable from all muscles tested, they were obtained from all participants for at least one muscle. Previous studies assessing the motor output patterns following complete SCI have revealed enlargement of single muscle representations for muscles innervated by nerve roots in proximity to the lesion level [1,6,7,23]. Our results are in line with these findings as cortical territory of APB, a muscle impaired in every participant, was enlarged compared to controls, while the territories of FCR and BB, muscles that likely retained a larger proportion of innervation (Table 1, see injury levels), did not differ in size from controls (Figure 4B). This is consistent with studies showing an increase in excitation of corticospinal pathways for muscles rostral to the spinal cord lesion [24]. Although the explanation for the expansion of muscle representation in SCI remains unclear, such changes may depend on use-dependency. Expansion occurs for muscles involved in motor skill learning [25,26,27], and for those essential for skilled sport performance [28,29]. We speculate that the expansion observed in the SCI group relative to controls may serve to maximize neural output to these muscles that, despite being functionally impaired, are integral to tasks of daily living (i.e., wheelchair operation). Alternatively, representational expansion may reflect reductions in spinal plasticity. It is suggested that impaired spinal plasticity in SCI underpins impairments in motor learning [30,31,32]. However, it is also known that progressive increases in M1 activation are observed in SCI during the first year following injury, reaching activation levels that are similar to controls [4]. Further, trans-synaptic upper motor neuron recruitment is impaired due to altered intracortical activation within M1 [33] and this may contribute to the expansion of cortical territory.

Changes in the location of these motor representational maps within the cortex were assessed by means of the CoG. We observed no changes in CoG _(AP)_ between our SCI and control groups. This is expected as the TMS-evoked representational locations are unlikely to be in the anterior-posterior direction as a reorganization of neighboring homuncular areas would be predominantly in the medio-lateral plane [34,35]. However, one previous study has shown a posterior shift in SCI [10], however this this discrepancy may relate to the muscles tested (extensor versus flexor forelimb muscle in the present study). Interestingly, we did observe differences in the CoG _(ML)_ component, indicating a medial shift in the upper limb muscle representations for the SCI group compared to the control group. This is the first TMS study to show a medio-lateral shift in SCI muscles. Previous work shows no somatotopic shifts in muscles, although this investigation was in acute SCI compared to chronic SCI studied here [7]. Additionally, one previous fMRI study reported no shift in the APB muscle representation, while the BB muscle was shifted medially in complete SCI participants and not incomplete SCI participants [36]. An explanation for the medial shift may relate to experience-dependent plasticity associated with the use of face and neck muscles to perform tasks previously performed by the hand. The increased usage of these alternative muscles may lead to an encroachment on cortical territory formerly occupied by hand/arm muscles. Future studies may investigate the possible expansion or medial shift of face/neck muscles.

Somatotopy was preserved in the dominant hemisphere for both SCI and controls; proximal muscles were located medial to distal muscles. This somatotopic distribution was not observed in the non-dominant hemisphere. Asymmetries between the left and right hemispheres on the basis of handedness have been shown using various neuroimaging modalities including cortical anatomy [37], greater area of hand activation assessed with magnetoencephalography (MEG) [38], and pattern of functional activation [39].

### Limitations

The method of TMS mapping was chosen to use an anchored grid that encompasses M1 and minimizing stimulation of associated cortical areas. However, it is possible that responses to TMS may be due to activation in surrounding associated motor regions, particularly at the edges of the grid space. Further, we include a sample size of 9 participants that, while exceeding the sample sizes of previous reports [1,6,7,8,9,10], is relatively small, limited mainly by the availability of participants. This small sample size has prevented us from drawing inferences about the relationship between factors including age, time since injury, physical activity, and rehabilitation program with changes in muscle representations. In addition, no information regarding the participant’s physical activity level or current rehabilitation program was obtained. However, it should be noted that these factors may play a role in modulating the functional changes that occur in motor cortex reorganization. Next, we mapped individuals with SCI who were taking their regular medications to control spasticity and pain. It remains unclear how these conditions and their pharmacological treatment may contribute to cortical reorganization following injury. Although baclofen does not influence TMS measures of RMT [40] or MEPs [41], we cannot determine the impact of the pharmaceutical treatment on cortical maps in our sample population. Withdrawal from pain and spasticity medication would impact the quality or the acquisition of data. Therefore, we need to consider that changes in brain maps may reflect neural plasticity associated with injury, experience, and medicinal management in SCI. Additionally, while we did not limit our population to right-handed dominance, we did ensure that our uninjured, control group was matched for handedness with our SCI group (see Table 1).

We did not assess the maximal force of the muscles that were mapped with TMS since the isolation of muscle force is technically challenging. Future studies should consider relating map parameters to muscle function with assessments of muscle force and/or central conduction time. Finally, we limited our SCI population to injury levels within the cervical spinal cord ranging from C3 to C8. All muscles tested were at or above the level of injury. Future, large-scale studies should attempt to differentiate between cortical maps of muscles at the level of injury versus cortical maps of muscles rostral to the injury level.

## 5. Conclusions

We report deviations in the M1 representation in individuals with SCI compared to uninjured controls that include a medial shift in representations and expansion of a hand muscle. Advances in our understanding of SCI are difficult due to the heterogeneous response to injury [3]. More reports are needed to further understand the plastic changes following SCI and how it impacts recovery of function.

## Figures and Tables

**Table 1:** Participant Demographics.

Sub	Years Since Injury	Injury Level	ASIA Score	Handedness (SCI)	Medications	Control Sub	Handedness (Controls)
1	4.5	C5–C6	C	L *	Fesoterodine	1	R
2	2	C6–C7	C	L	Diazepam, preganalin, cyclobenzaprine	9	L
3	39	C5	C	R	None	3	R
4	14	C6–C7	C	R	Baclofen	4	R
5	17	C4–C8	C	R	Botulinum toxin, Percocet	7	R
6	3	C4	C	R	Baclofen, pregabalin	6	R
7	2	C3–C4	C	R	Gabapentin, citalopram	8	R
8	33	C6–C7	C	R	Baclofen, clonazepam	4	R
9	3	C5	C	R	None	5	R

Note: ASIA scale = American Spinal Injury Association Impairment Scale; A = No sensory or motor function preserved in sacral segments; B = Sensory function is preserved with no motor function; C = Sensory function is preserved below the level of injury, most muscles below injury gave a grade less than 3; D = Motor function is preserved below the level of injury, most muscles below injury have a grade of 3 or more; E = Normal sensory and motor function. * indicates that this participant switched handedness following SCI. R = right, L = left, sub = subject, SCI = spinal cord injury.
